# Cecal Infusion of Sodium Propionate Promotes Intestinal Development and Jejunal Barrier Function in Growing Pigs

**DOI:** 10.3390/ani9060284

**Published:** 2019-05-28

**Authors:** Yanan Zhang, Huizi Chen, Weiyun Zhu, Kaifan Yu

**Affiliations:** 1 Laboratory of Gastrointestinal Microbiology, Jiangsu Key Laboratory of Gastrointestinal Nutrition and Animal Health, College of Animal Science and Technology, Nanjing Agricultural University, Nanjing 210095, China; ynzhang1515@163.com (Y.Z.); huizichen1994@163.com (H.C.); zhuweiyun@njau.edu.cn (W.Z.); 2 National Center for International Research on Animal Gut Nutrition, Nanjing Agricultural University, Nanjing 210095, China; 3 National Experimental Teaching Demonstration Center of Animal Science, Nanjing Agricultural University, Nanjing 210095, China

**Keywords:** propionate, intestinal development, tight junction, pigs

## Abstract

**Simple Summary:**

Microbial-derived short-chain fatty acids can exert influence on intestinal development and intestinal barrier function. Usually, it is well known that short-chain fatty acid butyrate provides energy for the colonic cell turnover and maintains the integrity of the colonic epithelium. However, the effect of short-chain fatty acid propionate on intestinal development and jejunal barrier function is given less attention. In this study, we found that cecal infusion of propionate promoted development of the jejunum and colon, and selectively enhanced jejunal tight junction protein expression. These results suggest that propionate by microbial fermentation in the hindgut has an important role in intestinal development and gut health.

**Abstract:**

Short-chain fatty acids (SCFAs) produced by microbial fermentation facilitate the differentiation and proliferation of intestinal epithelium. However, the role of individual SCFAs, such as propionate, on intestinal development is still unclear. In the present study, sixteen barrows fitted with a cecal fistula were randomly divided into two groups for cecal infusion of either saline (control group) or sodium propionate (propionate group). After 28 days, the length and the relative weight of intestinal segments were calculated, the intestinal morphology was assessed, and the expression of tight junction protein was measured using qPCR and Western blotting. Compared to the saline group, the length of the colon was significantly increased in the propionate group (*p* < 0.05). The jejunal villi length and villi/crypt ratio in the propionate group were significantly higher than in the saline group (*p* < 0.05). Furthermore, propionate infusion significantly upregulated the mRNA levels of *Claudin-4* and the expression of Claudin-1, Claudin-4, and Occludin protein in the jejunal mucosa (*p* < 0.05). Collectively, these findings revealed that the short-chain fatty acid propionate in the hindgut contributed to intestinal development, and selectively enhanced jejunal tight junction protein expression.

## 1. Introduction

The mammalian gastrointestinal tract harbors trillions of microorganisms. Thus, large amounts of small molecule metabolites are produced by intestinal microbiota, collectively termed the metabolome. Accumulating studies have demonstrated that intestinal microbial metabolites are linked to intestinal development and health [1,2,3], especially short-chain fatty acids (SCFAs). Short-chain fatty acids are the major products from microbial fermentation of non-digestive nutrients in the large intestine, including acetate, propionate, and butyrate. In general, SCFAs, known as energy substrates, are readily metabolized by intestinal epithelium and the liver. Furthermore, SCFAs serve as signaling molecules, playing key roles in the maintenance of intestinal barrier function. For example, SCFAs have been used to protect against intestinal inflammation [4], such as inflammatory bowel disease (IBD) [5].

SCFAs have been reported to be the major energy source for intestinal epithelial cells, especially butyrate, and provided 60–70% of the energy required for colonic epithelial development [6]. Further studies have shown that SCFAs influenced cellular processes and functions in the colon, such as cell proliferation and differentiation [7,8]. Previous studies reported that the intravenous or intracolonic infusion of SCFAs stimulated the growth of intestinal mucosa in rats [9,10]. Our previous study showed that intravenous infusion with sodium butyrate up-regulated the expression of genes related to intestinal development in normal growing pigs [11]. So far, however, most studies have focused on the effect of SCFA mixtures or individual butyrate on intestinal development [9,12]. Furthermore, limited information is available on the influence of propionate on intestinal development. Recently, SCFAs have been reported to activate intestinal gluconeogenesis via complementary mechanisms [8]. Thus, we speculated that propionate was also likely to exert beneficial effects on intestinal development.

The tight junction is one of the main junctional complexes in intestinal epithelial cells. It is composed of transmembrane proteins, such as Claudins and Occludin, and Zonula Occludens (ZOs). The integrity of the tight junction protects the intestinal epithelial barrier against the invasion of pathogens to maintain gut health. Some studies have reported that tight junction protein expression in the intestinal epithelium barrier was closely associated with SCFAs [13,14,15]. The administration of SCFA mixtures has been demonstrated to promote both protection and repair of intestinal epithelial barrier function in a Caco-2 cell model [16]. *In vivo*, the distal ileal infusion of SCFAs enhanced the expression of tight junction-related genes in the ileum and colon and improved the gut barrier function in a pig model [17]. Among the SCFAs, the role of butyrate in intestinal epithelial barrier function has been extensively explored. Dietary supplementation with sodium butyrate up-regulated the expression of *Claudin-3*, *Occludin*, and *ZO-1* genes in the colonic epithelium of weaning pigs [18] and the same effect was demonstrated *in vitro* [19]. Furthermore, propionate also showed benefits to gut barrier function. Oral administration of propionate enhanced colonic barrier function in rats [20]. Supplementation with propionate in the water ameliorated DSS (dextran sulfate sodium)-induced colitis by improving colonic barrier function in mice [21]. Therefore, these studies indicated that SCFAs maintained intestinal barrier function by modulating tight junction protein expression. Most previous studies have focused on the effects of SCFAs on colonic barrier function. However, the influence of propionate on small intestinal barrier function remains unclear.

The aim of the present study was to understand the trophic and healthy role of propionate in the gastrointestinal tract. We evaluated the effects of intra-cecal infusion of propionate on intestinal development and jejunal barrier function using a pig model with a cecal fistula. We assessed the length of intestinal segments, the intestinal index, and the intestinal morphology. In addition, the level of tight junction proteins in the intestine was detected by Western blotting.

## 2. Materials and methods

### 2.1. Experimental Procedures (Ethic)

The experimental proposal and procedures were approved by the Animals Care and Use Committee of Nanjing Agricultural University in compliance with Chinese guidelines for animal welfare (SYXK2017-0007). 

### 2.2. Animals, Experimental Design and Treaments

A total of 16 growing barrows (aged 52 days, weighted 16 ± 0.08 kg) (Duroc×Landrace×Large) from a commercial farm in Nanjing, Jiangsu Province, China, were selected and surgically installed with a fistula at the cecum. The pigs were installed with a T-fistula in the cecum by surgery according to previous methods [22,23,24]. The T-fistula was fabricated by industrial plastics (purchased from Chinese Academy of Agricultural Sciences, Beijing, China). The internal diameter, length, and wings of the T-fistula were 1.5, 8.2, and 10.0 cm, respectively. Before the surgery, the pigs were fasted for 12 h. During the surgery, the pigs were anesthetized by 5% isoflurane (95% oxygen) and were placed on a heating pad to maintain body temperature. After the surgery, the pigs were hypodermic injected with ceftriaxone sodium to protect against infections and pathogenic bacteria. After a 14-day convalescence, the pigs were randomly divided into two groups. The details of the grouping are as follows. Firstly, all pigs were numbered on the basis of body weight. Secondly, 16 different random numbers were selected from the table of random digits. Thirdly, the random numbers were sorted from small to large. Lastly, the front 8 numbers (corresponding to the front 8 pigs) were divided into the control group, the last 8 numbers (corresponding the last 8 pigs) were divided into the propionate group. Pigs were infused with either saline solution (0.9 wt.%, pH 5.8) (Control group, n = 8) or sodium propionate solution (2 M, pH 5.8) (Propionate group, n = 8). All the pigs were infused with 25 mL saline or propionic solution for one time point, twice per day at 7:00 am and 6:00 pm, respectively. The basal diet consisted of commercial concentrated feed and corn, and the ingredient and nutrient composition were shown in our pervious study [25]. Each pig was placed in an individual pen with a feeder and a low-pressure drinking nipple to provide ad libitum access to feed and water. During the 3 days pre-feeding and 28 days experimental period, the animal houses with excellent ventilation were regularly cleaned and room temperature was controlled at 24 ± 2 ℃. The material from this study and our previous study [25] are from the same experiment.

### 2.3. Slaughter Procedure and Sampling

At day 28, after 12 h fasting, the pigs were anesthetized by 5% isoflurane (95% oxygen) and slaughtered by jugular exsanguination. The gastrointestinal tract was removed immediately and intestinal segments were identified and ligated by using the following anatomical landmarks according to the previous method [26]. The stomach samples were collected from cardia to pylorus; the jejunum was collected from duodenal-jejunal junction to jejunal-ileal junction; the ileum was collected from jejunal-ileal junction to ileal-cecal junction (caudal third of intestine); the cecum was collected from cecal-ileal junction to cecal-colonic junction; and the colon was separated at rectal-anal junction. After the segments were separated, they were placed flat and stretched on the experimental desk. Then, the lengths of the jejunum, ileum, and colon were measured by a tapeline. Subsequently, the intestinal segments were cut open longitudinally, gently stripped of digesta, and cleaned with saline solution. Then, the weights of the stomach, ileum, cecum, and colon were recorded to calculate the intestinal index. The intestinal index was calculated by the formula: relative weight of intestine (%) = intestine weight/body weight × 100. For the histology analyses of the gut, the midpoints of the jejunum, ileum, and colon were located, and 2 cm tissue sections were obtained and then immediately fixed in 4 % polyformaldehyde solution. For intestinal mucosa samples, the intestinal mucosa was scraped off with a sterile glass microscope slide, collected in sterile microtubes, and immediately stored in liquid nitrogen for total RNA and protein isolation.

### 2.4. Intestinal Morphology Analysis

The intestinal tissues fixed in 4 % polyformaldehyde were dehydrated and embedded in paraffin. The tissues in paraffin block were cut into 4 μm slices and stained with hematoxylin and eosin (H&E staining). In H&E-stained tissues, nucleic acids stain dark blue and proteins stain red to pink or orange. The slices were photographed by a microscope (Nikon ECLIPSE BOi, Japan) and representative photographs of intestinal morphology were collected for the exhibition. The villi length (from tip to crypt mouth) and crypt depth (from crypt mouth to base) were measured using the Nis-Elements 4.20 software (Nikon, ECLIPSE BOi, Japan). There were 6 pigs in each group and 3 slides per pig for morphological characteristics analyses. Five well-oriented villus and crypts in each slide were measured.

### 2.5. RNA Isolation and Quantitative RT-PCR

Total RNA was extracted from the mucosa of jejunum and colon with Trizol kit (Takara Bio, Otsu, Japan) according to the manufacturer’s instruction. The concentration and purity of total RNA were analyzed using a NanoDrop spectrophotometer (ND-1000UV-Vis, Thermo Fisher Scientific, Waltham, MA, USA). The total RNA was reverse-transcribed to cDNA.

Quantitative RT-PCR was performed using SYBR Green qRT-PCR kit (Takara Bio, Otsu, Japan). Primers are listed in Table 1. The reaction conditions were as follows: 30 s at 95 ℃, 40 cycles at 95 ℃ for 5 s, 60 ℃ for 34 s, 95 ℃ for 15 s, 1 min at 60 ℃, and 15 s at 95 ℃. The β-actin served as the reference gene to correct the transcript levels of target genes, and fold expression levels of the genes of interest were calculated by 2^△△Ct^ method [27]. 

### 2.6. Total Protein Extraction and Western Blotting

Total protein was extracted from the intestinal samples using T-PER Tissue Protein Extraction Reagent (Thermo Pierce, NO.78510) and Halt Protease and Phosphatase Inhibitor Cocktail (100×) (Thermo Pierce, NO.78440). Briefly, the mucosae were homogenized by homogenizer in lysis buffer with a protease inhibitor and incubated on ice. Lysates were centrifuged at 10,000× *g* for 10 min at 4 ℃ and total protein concentration of the supernatant was determined with a BCA Protein Assay Kit (Beyotime, Shanghai, China). Total protein content of each sample was diluted with loading buffer and heated to 95 ℃ for 10 min. A total of 60 μg of protein was electrophoresed on an SDS-PAGE gel, and then the proteins were transferred onto polyvinylidene fluoride (PVDF) membrane (Millipore, Bedford, MA, USA) through electrophoresis. The membranes were blocked in Tris-buffered saline, including 0.05 % Tween-20 (TBS-T) and 5 % fat-free milk 1 h at room temperature and incubated with primary antibodies against ZO-1, Claudin-1, Claudin-4, Occludin (Abcam, Cambridge, UK), and β-actin (Santa Cruz, USA) overnight at 4 ℃. Following four washes with TBS-T, the membranes were incubated with secondary antibodies for 1 h at room temperature. After washing with TBS-T five more times, subsequent protein bands were visualized with SuperSignal West Dura Extended Duration Substrate, an enhanced chemiluminescence reagent (Thermo Pierce, USA). Band intensities were quantified with BandScan 5.0 software.

### 2.7. Statistical Analysis

All data were performed by statistical software SPSS Version 20.0 (SPSS Inc., Chicago, IL, USA) with the student’s *t*-test to compare the difference between the two groups. The individual pig regards the experimental unit used to access all the measurements, with 8 replications per group. Data are presented as mean ± standard error of the mean (SEM). Significant differences were declared when *p* value < 0.05.

## 3. Results 

### 3.1. The Intestinal Length and Intestinal Index

The lengths of the jejunum, ileum, and colon were measured and are shown in Table 2. The cecal infusion of propionate significantly increased the length of the colon by an average 1.13-fold compared to the saline group (*p* < 0.05). No significant differences were observed in the lengths of the jejunum and ileum between the propionate and saline infusion groups. The intestinal relative weight was calculated from the intestinal weight divided by the body weight and is presented in Table 3. In contrast, the relative weights of the stomach, ileum, cecum, and colon were not significantly different between the two groups. These results suggest that the cecal infusion of propionate promoted growth of the colon.

### 3.2. Intestinal Morphology

**Figure 1:**
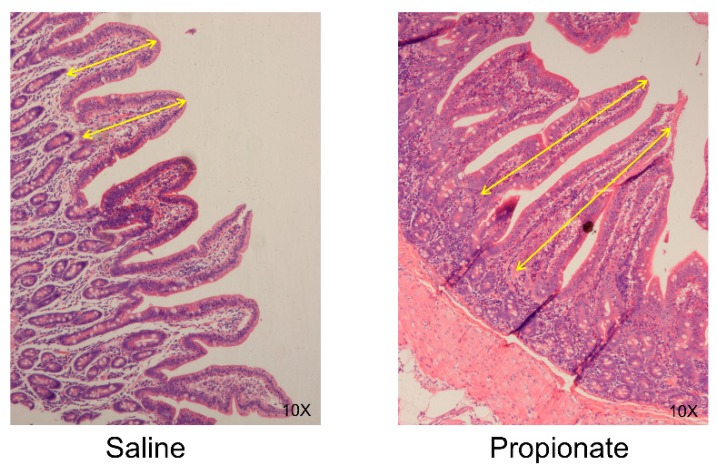
Histological evaluation of jejunal tissues from pig exposure to saline and propionate by hematoxylin and eosin (H&E)-Staining. The yellow arrows denote villi length used for measurement.

The morphological characteristics of jejunum were shown in Figure 1. The villi length, the crypt depth of the jejunum and ileum and the mucous membrane thickness and crypt depth of the colon were measured (Table 4). The jejunal villi length in the propionate group was significantly higher than in the saline group by an average 1.44-fold (*p* < 0.05). There was no obvious difference in the crypt depth of the jejunum between the groups, while propionate significantly increased the villi/crypt ratio (*p* = 0.04) (Table 4). In the ileum, the cecal infusion of propionate did not alter the villi length, crypt depth, or villi/crypt ratio (Table 4). The mucosal membrane thickness and crypt depth were also unchanged in the colon of the propionate group (Table 4). Therefore, these data suggest that the cecal infusion of propionate potentially improved the development of epithelium in the jejunum.

### 3.3. Tight Junction Expression

**Figure 2:**
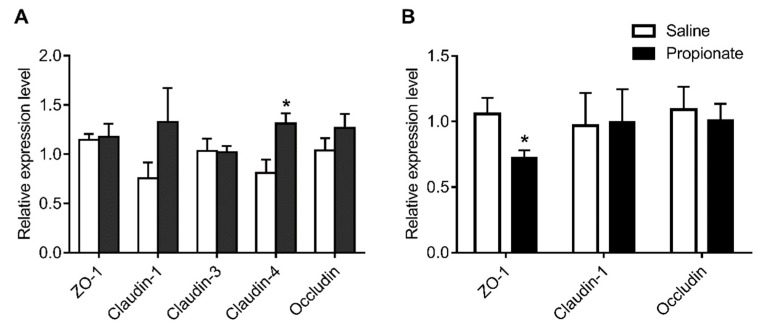
Effect of the cecal infusion of propionate on the mRNA levels of tight junction proteins in the jejunum (**A**) and colon (**B**).

**Figure 3:**
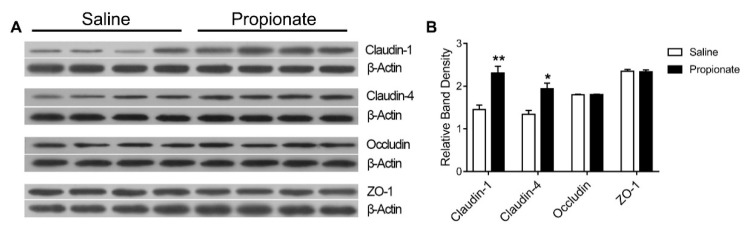
Effect of the cecal infusion of propionate on the expression of tight junction protein expression in the jejunum: (**A**) the bands and (**B**) relative band density.

The expression of genes related to the tight junction in the intestinal epithelium was determined by quantitative RT-PCR (Figure 2). Propionate administration upregulated *Claudin-4* expression (*p* < 0.05), while the expression of *ZO-1*, *Claudin-1*, *Claudin-3*, and *Occludin* was unchanged in the jejunum (*p* > 0.05) (Figure 2A). In addition, the expression of colonic genes related to the tight junction, including *Claudin-1* and *Occludin*, was unaltered, except for the down-regulation of *ZO-1* (*p* < 0.05) (Figure 2B). The effect of cecal infusion of propionate on the expression of jejunal tight junction proteins is shown in Figure 3. The protein levels of Claudin-1 and Claudin-4 in the propionate group were significantly higher than in the saline group (*p* < 0.05) (Figure 3). However, cecal infusion of propionate had no significant influence on the protein expression of Occludin and ZO-1 (Figure 3). Therefore, the results showed that propionate infusion selectively enhanced jejunal tight junction protein levels to maintain intestinal barrier function.

## 4. Discussion

In the present study, we used a fistula pig model to examine the effects of propionate on intestinal development and intestinal barrier fuction. We found that the cecal infusion of propionate promoted colon development and enhanced the expression of tight junction protein in the jejunum. Collectively, the results demonstrated that propionate in hindgut played a positive role in intestinal development and intestinal barrier function.

Short-chain fatty acids serve as one of the substrates for energy metabolism and intestinal growth. The effects of SCFAs have been found to be dose-dependent and differ among various short-chain fatty acids (in the order of butyrate > propionate > acetate) [31]. Previous studies have reported that butyrate was the major energy source for colonic epithelium cell turnover [32,33], while propionate and acetate were absorbed into the bloodstream and transported to hepatic cells to modulate lipid and glucose metabolism [34]. However, in the current study, the cecal infusion of propionate increased the length of the colon, suggesting that propionate also had a crucial role in colonic epithelial growth. Consistently, cecal propionate infusion decreased the colonic butyrate concentration, which suggested that less butyrate was required as an energy source [25]. Several studies have demonstrated that SCFAs absorbed by the intestinal mucosa were converted to ketone bodies to supply energy for the differentiation and proliferation of colonic cells [6,35,36].

Interestingly, the present study showed that the cecal infusion of propionate increased the jejunal villi length and the villi/crypt ratio, which indicated that propionate in the hindgut influenced the morphology of the jejunal epithelium. A previous study reported that the trophic effect of colonic infusion with SCFAs on the jejunum of rats was accomplished via a mechanism involving the autonomic nervous system [37]. Therefore, the trophic effect of propionate on the jejunum was probably mediated through the gut-brain axis. A further study demonstrated that SCFAs likely stimulated the secretion of gastrin from the jejunal endocrine G cells via the gut-brain axis in rats, which was beneficial to villi growth and crypt depth in the jejunum [38]. These studies demonstrated that SCFAs could stimulate the production of nerve signals from the colon to the central nervous system via the autonomic nervous system. Subsequently, a secondary nerve signal from the central nervous system was delivered to the intestine, which stimulated the secretion of hormones and growth factors from the intestinal epithelial cells to promote the development of intestinal morphology [38]. Therefore, we presume that the cecal infusion of propionate regulated the development of jejunum through the same mechanism, although further study is necessary. Furthermore, SCFAs may affect jejunal development through liver metabolic pathways. Besides the intestinal epithelium, the liver is a main site for SCFA metabolism. Short-chain fatty acids are converted to a variety of metabolites in the liver, including ketone bodies (acetoacetic acid, β-hydroxybutyric acid), glutamate, and glutamine, which serve as nutritional substrates for the growth of intestinal mucosa [39,40]. Previous studies have indicated that ketone bodies and glutamate were the main fuel sources for oxidative metabolism in the small intestine, which supplies energy for gut growth [41,42]. Hence, in the present study, the cecal infusion of propionate may have indirectly provided the energy for jejunum mucosal growth by influencing hepatic metabolism.

However, in this study, the villi length and crypt depth of the ileum were not altered. Reilly and colleagues reported that SCFAs likely stimulated the secretion of gastrin from endocrine G cells via the gut-brain axis in rats, and gastrin plays a positive role in promoting the intestinal epithelial development [38]. Some studies demonstrated that the distribution of gastrin cells in the gastrointestinal tract is different [43,44]. Therefore, the difference of gastrin cell distribution in the intestinal tract may be one of the reasons why cecal infusion with propionate has little effect on the morphological characteristics of ileum, which needs further investigation.

As a part of the intestinal epithelial barrier, tight junction proteins play an important role in maintaining intestinal health by regulating epithelial permeability and resisting the invasion of pathogenic bacteria. Previous studies have shown that SCFAs had a positive role in maintaining intestinal barrier function [15,45]. In the current study, cecal infusion with propionate up-regulated the *Claudin-4* gene expression and Claudin-1, Claudin-4, and Occludin protein levels in the jejunal mucosa. In vitro, different levels of butyrate upregulated levels of the tight junction protein in IPEC-J2 cells through the activation of Akt/mTOR signaling pathways [19], consistent with this study. However, cecal infusion with propionate had no significant effect on the tight junction gene expression in the colon. These results are inconsistent with the previous study by Xia et al. [20], which might be caused by different physiological statuses of animal models. The administration of propionate to normal lactating rats increased the expression of *ZO-1*, *Claudin,* and *Claudin-8* in the colonic mucosa by activating ERK1/2 and p38MAPK signaling pathways, which enhanced intestinal barrier function [20]. In addition, a previous study showed that propionate inhibited the expression of *ZO-1* and *Occludin* and improved the intestinal barrier function in mice with DSS-induced colitis [21]. These studies revealed that SCFAs maintained intestinal barrier integrity by regulating the expression of tight junction proteins through different mechanisms.

## 5. Conclusions

In this study, by using a fistula pig model, the present study demonstrated that cecal infusion with propionate increased colonic length and selectively enhanced the levels of tight junction proteins in the jejunal mucosa. These findings highlight the positive role of propionate in the large intestine on the intestinal development and epithelial barrier function in growing pigs.

## Figures and Tables

**Table 1:** Lists of primers used in this study.

Genes	Forward Primer (5’-3’)	Reverse Primer (5’-3’)	Reference
β-actin	F:CCACGAAACTACCTTCAACTC	R:TGATCTCCTTCTGCATCCTGT	[28]
ZO-1	F: GAGGATGGTCACCGTGGT	R: GGAGGATGCTGTTGTCTCGG	[29]
Claudin-1	F: AGATTTACTCCTACGCTGGT	R: GCACCTCATCATCTTCCAT	[30]
Claudin-3	F:CCTACGACCGCAAGGACTAC	R:GACTGGTCTCGGATGCAAGG	[30]
Claudin-4	F:CGTACCGACAAGCCCTACTC	R:GCAGTCCAGGGAGAAACCAA	[30]
Occludin	F:ATGCTTTCTCAGCCAGCGTA	R:AAGGTTCCATAGCCTCTCGGTC	[29]

**Table 2:** Effects of the cecal infusion of sodium propionate on the length of different intestinal segments.

Item	Saline	Propionate	*p*-Value
Jejunum (cm)	752.50 ± 49.05	795.63 ± 47.69	0.54
Ileum (cm)	719.38 ± 65.67	712.50 ± 71.21	0.94
Colon (cm)	227.50 ± 8.40	258.13 ± 8.45	0.02

**Table 3:** Effects of the cecal infusion of sodium propionate on the intestinal index.

Item	Saline	Propionate	*p*-Value
Relative weight of stomach (%)	0.66 ± 0.02	0.68 ± 0.03	0.54
Relative weight of Ileum (%)	1.61 ± 0.12	1.52 ± 0.14	0.60
Relative weight of cecum (%)	0.23 ± 0.03	0.19 ± 0.01	0.25
Relative weight of colon (%)	1.29 ± 0.07	1.35 ± 0.08	0.57

**Table 4:** Effects of the cecal infusion of sodium propionate on the intestinal morphology.

Item	Saline	Propionate	*p*-Value
Jejunum			
Villi length (µm)	365.56 ± 24.87	524.75 ± 40.95	0.03
Crypt depth (µm)	207.18 ± 54.58	170.03 ± 11.07	0.57
Villi/Crypt	1.80 ± 0.50	3.10 ± 0.40	0.04
Ileum			
Villi length (µm)	355.79 ± 83.27	456.43 ± 13.41	0.44
Crypt depth (µm)	132.51 ± 53.40	154.95 ± 35.61	0.74
Villi/Crypt	3.50 ± 2.04	3.41 ± 1.02	0.97
Colon			
Mucosa thickness (µm)	439.20 ± 30.83	423.24 ± 11.13	0.64
Crypt depth (µm)	204.42 ± 21.42	168.70 ± 25.00	0.30
